# Metabolic Alterations Associated with Early-Stage Hepatocellular Carcinoma and Their Correlation with Aging and Enzymatic Activity in Patients with Viral Hepatitis-Induced Liver Cirrhosis: A Preliminary Study

**DOI:** 10.3390/jcm9030765

**Published:** 2020-03-12

**Authors:** Chung-Man Moon, Sang Soo Shin, Suk Hee Heo, Yong Yeon Jeong

**Affiliations:** 1 Quantitative Medical Imaging Section, National Institute of Biomedical Imaging and Bioengineering, National Institutes of Health, Bethesda, MD 20892, USA; chmanmoon2@gmail.com; 2 Research Institute of Medical Sciences, Chonnam National University, Gwangju 61469, Korea; 3 Department of Radiology, Chonnam National University Medical School, Gwangju 61469, Korea; sheo@jnu.ac.kr (S.H.H.); yjeong@jnu.ac.kr (Y.Y.J.); 4 Department of Radiology, Chonnam National University Hospital, Gwangju 61469, Korea; 5 Department of Radiology, Chonnam National University, Hwasun Hospital, Hwasun 58128, Korea

**Keywords:** liver cirrhosis, hepatocellular carcinoma, spectroscopy, aging, enzymatic activity

## Abstract

Liver cirrhosis (LC) can develop hepatocellular carcinoma (HCC). However, noninvasive early diagnosis of HCCs in the cirrhotic liver is still challenging. We aimed to quantify the hepatic metabolites in normal control (NC), cirrhotic liver without HCC, cirrhotic liver with HCC (CLH), and early-stage HCC groups using proton magnetic resonance spectroscopy (^1^H-MRS) with a long echo-time (TE) and to assess the potential association between the levels of hepatic metabolites in these four groups and aging and enzymatic activity. Thirty NCs, 30 viral hepatitis-induced LC patients without HCC, and 30 viral hepatitis-induced LC patients with HCC were included in this study. ^1^H-MRS measurements were performed on a localized voxel of the normal liver parenchyma (*n* = 30) from NCs, cirrhotic liver parenchyma (*n* = 30) from LC patients without HCC, and each of the cirrhotic liver parenchyma (*n* = 30) and HCC (*n* = 30) from the same patients in the CLH group. Generalized estimating equations were used to evaluate potential risk factors for changes in metabolite levels. Potential associations between metabolite levels and age and serum enzymatic activities were assessed by correlation analysis. The levels of lactate+triglyceride (Lac+TG) and choline (Cho) in HCC were significantly higher compared to those in LC and CLH. A potential risk factor for changes in the Lac+TG and Cho levels was age, specifically 60–80 years of age. In particular, the Lac+TG level was associated with a high odds ratio of HCC in males aged 60–80 years. The Lac+TG and Cho concentrations were positively correlated with lactate dehydrogenase and alkaline phosphatase activities, respectively. Our findings suggested that ^1^H-MRS measurement with a long TE was useful in quantifying hepatic Lac+TG and Cho levels, where higher Lac+TG and Cho levels were most likely associated with HCC-related metabolism in the viral hepatitis-induced cirrhotic liver. Further, the level of Lac+TG in HCC was highly correlated with older age and lactate dehydrogenase activity.

## 1. Introduction

Liver cirrhosis (LC) secondary to chronic hepatitis can lead to serious complications. In severe cases, cirrhotic patients can develop hepatocellular carcinoma (HCC), which is the most common primary hepatic malignant neoplasm. Therefore, monitoring disease progression is clinically important [1,2]. Moreover, even though the incidence of HCC tends to increase with an age effect, relatively few studies [3,4] to date have investigated its association with age. Although a previous etiological study [5] reported that hepatitis viruses, alcohol consumption, and aflatoxin may be the main risk factors for HCC, the genotypic and phenotypic features associated with these risk factors in HCC are greatly diverse. In addition, mortalities from HCC are mainly due to hepatitis B and C virus infections, which explained 78.5% of HCC-induced mortality in 2013 [6].

Although hepatocellular carcinogenesis in patients with LC largely appears to be a continuous, multistep differentiation process from a benign regenerative nodule to HCC [7], early diagnosis of HCCs is still challenging [8]. Liver biopsy is the gold standard for diagnosing chronic liver disease; however, its use is limited due to its invasiveness, as well as sampling bias and interobserver variability [9]. Several imaging techniques utilizing ultrasound (US), computed tomography (CT), and magnetic resonance imaging (MRI) represent noninvasive alternatives to liver biopsy and can potentially be used for the early detection and localization of HCCs as well as differential diagnosis between overt HCCs and cirrhosis-related borderline lesions [10]. However, the development of advanced noninvasive diagnostic methods, with an ability to diagnose HCCs at an earlier stage, is of clinical importance and expected to reduce HCC-related mortality.

Proton magnetic resonance spectroscopy (^1^H-MRS) is a noninvasive imaging technique that provides a method for biochemical characterization of normal and abnormal tissues, both in vitro and in vivo [11]. ^1^H-MRS has been used not only to evaluate liver function in diffuse hepatic diseases, such as liver steatosis, hepatitis, and cirrhosis, but also to distinguish between benign and malignant hepatic tumors [10]. Although ^1^H-MRS has been broadly applied to the central nervous system, breast, and prostate, and has demonstrated its value in diagnosing malignancies, the use of ^1^H-MRS in the liver has been relatively understudied. To our knowledge, only a small number of studies have used a clinical MR scanner to assess the metabolic status in LC and HCC using ^1^H-MRS with a short echo-time (TE) [12]. Most of these studies, however, have focused on comparing metabolite levels between tumors in patients with HCC and cirrhotic liver in patients without HCC. Since the molecular and biological characteristics of liver diseases are variable [10], it is important to acquire and compare in vivo ^1^H-MRS data from cirrhotic liver with HCC (CLH) and HCC in the same patient. Moreover, mechanisms underlying the formation of and change in cellular metabolites are impacted by aging and enzymatic activity in patients with LC, CLH, and HCC. In contrast to the short TE ^1^H-MRS, ^1^H-MRS with a long TE is useful in differentiating metabolites with long T2 relaxation times, such as lactate (Lac) and choline (Cho), as reported in a previous in vivo ^1^H-MRS study [9]. We postulated that the use of long TE ^1^H-MRS would clarify the metabolic changes caused by cirrhotic liver and HCC.

In this study, we aimed to measure and compare hepatic metabolites in normal control (NC), cirrhotic liver without HCC, CLH, and early-stage HCC groups, and investigate their associations with aging and enzymatic activity using in vivo ^1^H-MRS with a long TE. In addition, we evaluated the diagnostic performance of ^1^H-MRS for the assessment of HCC resulting from a cirrhotic liver.

## 2. Materials and Methods 

### 2.1. Study Design and Patient Population

Before initiating the study, the institutional review board of Chonnam National University Hospital approved the study protocol (CNUH-2017-158), and a written informed consent was provided by all patients. All methods were performed in accordance with the relevant guidelines and regulations of the institution.

In this prospective study, patients were enrolled between July 2017 and June 2018. Based on a preliminary study, it was estimated that at least 30 patients were required for each group in order to significantly quantify the differences in hepatic metabolites among different groups with an α error of 0.05 and a β error of 0.2.

One hundred and ninety-four consecutive patients underwent ^1^H-MRS. Among these patients, 104 patients were excluded based on the following criteria: (1) HCC less than 2 cm in the longest diameter (*n* = 42); (2) fatty deposition in the liver (*n* = 17); (3) patients who did not provide written informed consent (*n* = 22); (4) alcohol as a cause of LC (*n* = 18); and (5) patients who did not undergo MRI examination due to poor respiratory cooperation (*n* = 4) and claustrophobia (*n* = 1). The final inclusion criteria for enrollment were as follows: (1) aged 20 years or older; (2) absence of fatty liver; (3) absence of a history of liver disease in the NC group; (4) hepatitis B and/or C virus infection as a cause of LC; and (5) early-stage HCC (a single tumor <5 cm in size or three tumors that are each <3 cm in size, with no evidence of portal invasion and extrahepatic spread). Therefore, in patients with HCC, the size of HCC ranged from 2 cm to 5 cm [13].

The absence of fatty liver was defined as less than 5% based on MR imaging using a high-speed T2-corrected multi-echo (HISTO) MRS. All patients underwent a physical examination, laboratory tests, CT, and MR imaging to confirm the presence or absence of liver disease. The diagnosis of LC was made based on the results of the liver biopsy or radiological findings, which included morphologic changes in the liver (hepatic surface nodularity and segmental atrophy or hypertrophy) and stigmata of portal hypertension (splenomegaly, portosystemic collaterals, and ascites) [8]. HCC was confirmed by core needle biopsy or typical radiological findings (arterial phase hyper-enhancement and delayed washout) [14]. In patients with more than one HCC, ^1^H-MRS data were acquired from the largest HCC.

Consequently, this prospective study consisted of 30 NCs, 30 cirrhotic patients without HCC, and 30 cirrhotic patients with HCC. In cirrhotic patients, the causes of LC were hepatitis B virus (*n* = 41), hepatitis C virus (*n* = 12), and both hepatitis B and C viruses (*n* = 7). In the 30 cirrhotic patients with HCC, 18 patients had a single HCC, while 12 patients had more than one HCC. The mean size of HCCs was 3.1 ± 0.32 (range 2.4–4.7) cm. LC was established histologically (*n* = 19) or radiologically (*n* = 41), while HCC was diagnosed based on histological (*n* = 9) or imaging (*n* = 21) findings. The locations of HCC were segment II (*n* = 3), segment III (*n* = 2), segment IV (*n* = 3), segment V (*n* = 4), segment VI (*n* = 6), segment VII (*n* = 4), and segment VIII (*n* = 8).

### 2.2. Serum Biochemical Analysis

From all patients before MRI examination, blood was collected using BD Vacutainer SST II Advance tubes (Cat no. 368640; Becton, Dickinson and Company, UK) containing gel separator and silica clot activator. Serum was separated after 30 min of incubation at room temperature followed by centrifugation for 15 min at 3500 RPM. All biomarkers for liver function test were examined on routine chemistry analyzers AU5800 (Beckman Coulter, Inc., CA, USA) or ADVIA 1800 (Siemens, NY, USA). For AU5800, the reagents were used as follows: aspartate aminotransferase (AST), Cat no. OSR6109; alanine aminotransferase (ALT), Cat no. OSR6107; lactate dehydrogenase (LDH), Cat no. OSR6126; alkaline phosphatase (ALP), Cat no. OSR6204; Glucose, Cat no. OSR6221; and triglyceride (TG), Cat no. OSR61118 (Beckman Coulter, Inc.). For ADVIA 1800, the reagents provided by same manufacturer were used as follows: AST, Cat no. 03039631; ALT, Cat no. 03036926; LDH, Cat no. 03030863; ALP, Cat no. 10916067; Glucose, Cat no. 05001429; and TG, Cat no. 10335892 (Siemens).

### 2.3. MR Imaging and Spectroscopy

All patients fasted at least 8 h prior to the MRI examination. MR data were acquired using a 3-T MR scanner (Magnetom TimTrio, Siemens Healthcare, Erlangen, Germany), with a 32-channel receiver body matrix coil. Sagittal, coronal, and axial images were acquired to provide the reference anatomic images for voxel localization in ^1^H-MRS. Together with the segmented breath-hold method, the T1-weighted images (TR/TE = 3.3/1.2 msec) were acquired with 3D T1 high-resolution isotropic volume excitation pulse sequence with field of view (FOV) = 37 × 40 × 14 cm^3^; matrix size = 512 × 512; number of excitation (NEX) = 2; slice thickness = 0.74 × 0.74 × 2.0 mm^3^; number of slices = 70; and scan time = 16 sec. In this study, a high-speed T2-corrected multi-echo (HISTO) MRS for quantification of hepatic steatosis was performed with a stimulated echo acquisition mode sequence with the following parameters: TR = 3000 ms; mixing time = 10 ms; and 5 TEs = 12, 24, 36, 48, and 72. A total of 2048 points were acquired at a bandwidth of 3000 Hz. Data acquisition was performed within a single breath hold with a voxel size of 2 × 2 × 2 cm^3^, which was in the region of interest (ROI) voxels of ^1^H-MRS.

**Figure 1:**
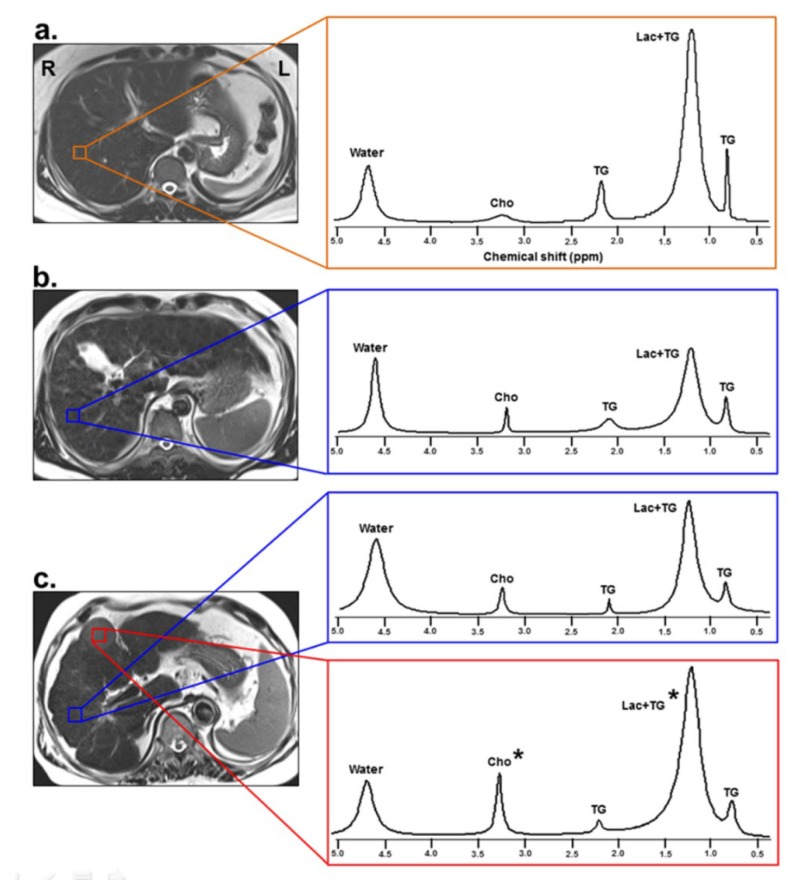
An axial plane anatomic image with spectroscopy voxel localized in the right lobe of the liver (left) and representative ^1^H MR spectra (right) from NC (**a**), LC patient without HCC (**b**), and LC patient with HCC (**c**). The voxel is targeted to the normal liver (brown), cirrhotic liver (blue), and HCC (red). The asterisk (*) indicates a significant difference in the metabolic concentration ratios among the groups (*p* < 0.05). R, right; L, left; NC, normal control; LC, liver cirrhosis; HCC, hepatocellular carcinoma; Lac+TG, lactate+triglyceride; Cho, choline; TG, triglyceride.

In order to detect Lac and Cho metabolites with a long T2 relaxation time, the single-voxel ^1^H-MRS measurements were performed using a point-resolved spectroscopy sequence (PRESS) with the following parameters: TR/TE = 2000/288 msec; six acquisitions within a single breath hold; 2000 Hz spectral width; and 1024 data points [9]. Six saturation bands were placed around the voxel to diminish tissue contamination from the adjacent structures. To maintain position consistency, one radiologist, who had 16 years of experience in abdominal radiology, positioned all MRS voxels. In the NC and LC groups, a 2 × 2 × 2 cm^3^ voxel was placed on the right lobe of the liver (Figure 1). In the HCC group, one voxel (2 × 2 × 2 cm^3^) was placed within HCC and another voxel (2 × 2 × 2 cm^3^) was placed on the cirrhotic liver parenchyma (Figure 1). In particular, when localizing a voxel within the HCC, care should be taken to avoid necrotic and hemorrhagic sites, in order to minimize the effects of intra-tumor heterogeneity of the HCC. Data acquisition started when the water suppression level was over 90% and bandwidth was below 10 Hz after auto-shimming. A compression belt was used to reduce respiratory motion artifacts. Intermittent breath hold was adopted during data acquisition, starting at end-expiration. Based on the monitoring of patients’ breathing, data acquisition was stopped earlier or whenever the patient had to breathe again.

### 2.4. MR Spectra Analysis

The HISTO spectra were analyzed using an MRS data analysis package (Siemens Medical Solutions). At each TE, the water (4.7 parts per million (ppm)) and fat (0.9, 1.3, 2.1, and 2.75 ppm) signals were measured. Each measurable peak area was individually corrected for the T2 decay using non-linear least-square fitting to determine their relative proton densities. The relative proton densities of the fat peaks located underneath the water peaks were determined using the method described by Hamilton et al. [15]. The total proton density was defined as the sum of all T2-corrected individual fat peaks. The proton density fat fraction (FF) (%) was calculated as the ratio of the fat proton density to the sum of the fat and water proton densities.

MR spectra were analyzed using a Java-based MR user interface software (jMRUI version 4.0; developed by A. van den Boogaart, Katholieke Universiteit Leuven, Leuven, Belgium). The free induction decay data were apodized by Gaussian broadening function with 10 Hz, zero-filled to 4096 data points, and Fourier-transformed to frequency domain. Zero- and first-order phase correction was applied to each spectrum signal. Major hepatocellular metabolites were assigned as TG at 0.9 and 2.1 ppm, Lac+TG at 1.3 ppm, and Cho at 3.2 ppm. All spectra were fitted in the time domain using a non-linear least-squares algorithm in the jMRUI software package (AMARES). The residual water peak at 4.7 ppm was used as an internal reference, and its frequency shifted to 4.7 ppm for the quantification of the hepatic metabolites. The ratios of metabolites relative to water were calculated using the heights of each metabolite.

### 2.5. Statistical Analysis

The differential metabolite levels and serum biochemistry parameters among the four groups were analyzed using analysis of covariance (ANCOVA), with adjustments for age and sex, with Tukey’s post-hoc test at *p* < 0.05. To identify the reliable measurements of metabolites, the intraclass error among in vivo ^1^H-MRS measurements was assessed using the intraclass correlation coefficient (ICC). Further, the associations of metabolite levels with serum enzymatic activities and age were assessed by Pearson’s correlation. In addition, generalized estimating equations (GEE) were used to evaluate potential risk factors for changes in metabolite levels adjusting for clustering effects in each patient. Also, we performed a receiver operating characteristics (ROC) curve analysis to evaluate the diagnostic performance of long TE ^1^H-MRS data in LC, CLH, and HCC.

## 3. Results 

### 3.1. Comparison of Serum Enzymatic Activities

There were significant differences in serum enzymatic activities for AST, LDH, glucose, and ALP among NCs, LC patients without HCC, and LC patients with HCC (Table 1). The AST, LDH, and glucose levels were significantly different between the NC and LC with HCC groups. In addition, the ALP level was significantly different between the NC and LC groups. There was a significant difference in the glucose level between the LC and HCC groups. No other significant differences were found in the remaining enzymes among the patients.

### 3.2. Comparison of the Degree of Steatosis and Hepatocellular Metabolite Levels for Lac+TG, Cho, and TG

Regarding the degree of steatosis, the FF (%) (NC vs. LC vs. CLH vs. HCC = 4.2 ± 2.3 vs. 4.6 ± 2.5 vs. 4.5 ± 2.2 vs. 3.1 ± 2.2) was not significantly different among the four groups.

Figure 1 shows representative ^1^H-MR spectra for the NC, LC, CLH, and HCC groups. The average ICC was 0.987 (95% confidence interval (CI) = 0.982–0.990) (*p* < 0.001) for Lac+TG, 0.981 (95% CI = 0.975–0.986) (*p* < 0.001) for Cho, 0.998 (95% CI = 0.997–0.998) (*p* < 0.001) for TG at 0.9 ppm, and 0.986 (95% CI = 0.980–0.991) (*p* < 0.001) for TG at 2.1 ppm.

**Figure 2:**
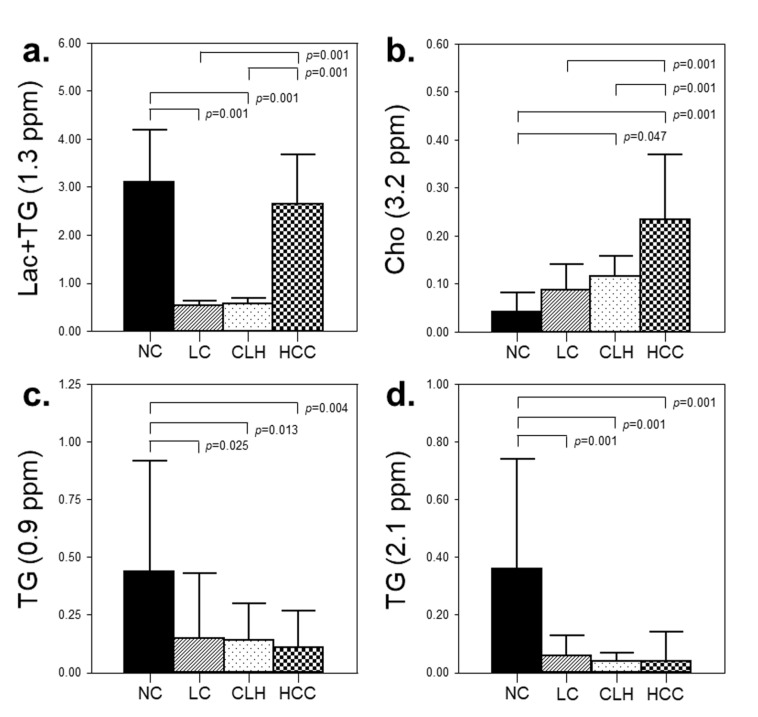
The quantification of the hepatic metabolites for Lac+TG (1.3 ppm) (**a**), Cho (3.2 ppm) (**b**), TG (0.9 ppm) (**c**), and TG (2.1 ppm) (**d**) were evaluated as the relative values to the residual water peak (4.7 ppm). The differential metabolite levels among the four groups were analyzed using analysis of covariance (ANCOVA), with adjustments for age and sex, with Tukey’s post-hoc test at *p* < 0.05. NC, normal control; LC, liver cirrhosis without HCC; CLH, cirrhotic liver with HCC; HCC, hepatocellular carcinoma; Lac+TG, lactate+triglyceride; Cho, choline; TG, triglyceride.

Hepatocellular metabolites exhibited distinct patterns among different groups (Figure 2). The levels of Lac+TG and Cho in the HCC group were significantly higher compared to those in the LC and CLH groups (*p* < 0.05). In contrast, similar levels of TGs (0.9 and 2.1 ppm) were observed between the LC, CLH, and HCC groups. The LC and CLH groups exhibited similar levels of Lac+TG and Cho.

### 3.3. Correlation of Lac+TG and Cho Levels with Enzymatic Activities of LDH and ALP and Age

**Figure 3:**
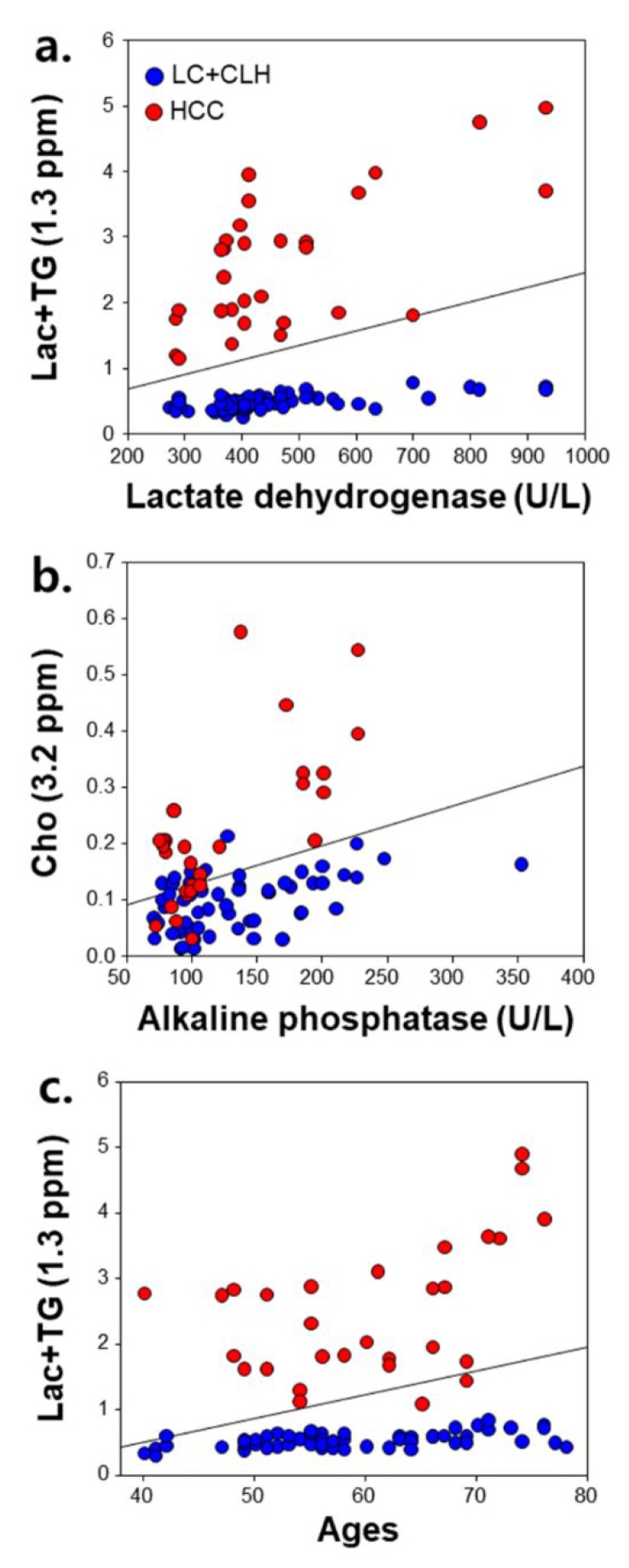
Correlations of metabolite concentrations with enzymatic activities and age in LC and CLH (blue), and HCC (red) groups. Lac+TG and Cho concentrations were positively correlated with lactate dehydrogenase activity (γ = 0.299, *p* = 0.004) (**a**) and alkaline phosphatase activity (γ = 0.338, *p* = 0.001) (**b**), respectively. Lac+TG concentration was also positively correlated with age (γ = 0.316, *p* = 0.003) (**c**). LC, liver cirrhosis without HCC; CLH, cirrhotic liver with HCC; HCC, hepatocellular carcinoma; Lac+TG, lactate+triglyceride; Cho, choline.

Figure 3 shows the correlations of the hepatic metabolites with enzyme levels and age in the LC, CLH, and HCC groups. The Lac+TG and Cho levels were positively correlated with LDH activity (γ = 0.299, *p* = 0.004) (Figure 3a; Table 2) and ALP activity (γ = 0.338, *P* = 0.001) (Figure 3b; Table 2) in a combination of the LC, CLH and HCC groups, respectively. In addition, the Lac+TG concentration was positively correlated with age (γ = 0.316, *p* = 0.003) (Figure 3c). However, there were no significant correlations between the NC group and other enzyme levels.

### 3.4. GEE Analysis for Risk Factors Associated with Metabolic Changes of Lac+TG and Cho

A potential risk factor for changes in the Lac+TG and Cho levels was 60–80 years of age (odds ratio (OR) = 1.508, *p* = 0.009 and OR = 1.049, *p* = 0.024, respectively). Given the interaction effect, the Lac+TG level was associated with higher OR of HCC in males and females aged between 40 and 80 years, especially in males aged between 60 and 80 years (OR = 15.184, *p* = 0.001), relative to the OR of LC and CLH in males and females aged between 60 and 80 years. The Cho level was also associated with HCC in males and females aged between 60 and 80 years, as shown in Table 3.

### 3.5. ROC Analysis for the Diagnosis of Early-Stage HCC using Lac+TG and Cho Levels 

**Figure 4:**
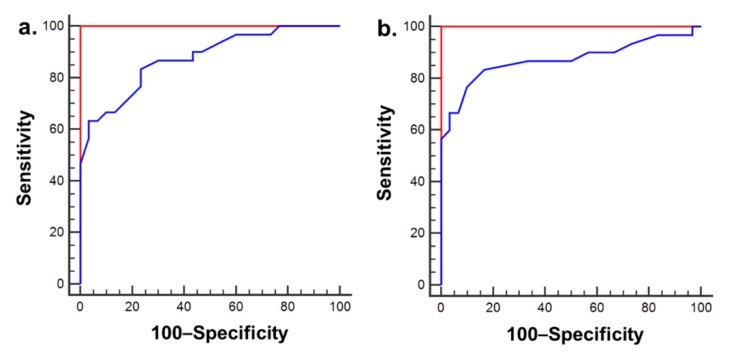
ROC curves of Lac+TG (red) and Cho (blue) metabolite concentration ratios for the differentiation between LC and HCC (**a**), and CLH and HCC (**b**). The AUC to distinguish HCC from LC was 1.00 (95% CI 0.94–1.00) (*p* < 0.001) for Lac+TG and 0.88 (95% CI 0.76–0.95) (*p* < 0.001) for Cho. Also, the AUC to distinguish HCC from CLH was 1.00 (95% CI 0.94–1.00) (*p* < 0.001) for Lac+TG and 0.87 (95% CI 0.76–0.94) (*p* < 0.001) for Cho. LC, liver cirrhosis without HCC; CLH, cirrhotic liver with HCC; HCC, hepatocellular carcinoma; Lac+TG, lactate+triglyceride; Cho, choline.

The area under the curve (AUC) to distinguish HCC from LC was 1.00 (95% CI 0.94–1.00) (*p* < 0.001) for Lac+TG and 0.88 (95% CI 0.76–0.95) (*p* < 0.001) for Cho. The diagnostic accuracy had a sensitivity and specificity of 100% at a cut-off value of 0.99 for Lac+TG, and sensitivity and specificity of 63% and 97%, respectively, at a cut-off value of 0.13 for Cho (Figure 4a). Meanwhile, the AUC to distinguish HCC from CLH was 1.00 (95% CI 0.94–1.00) (*p* < 0.001) for Lac+TG and 0.87 (95% CI 0.76–0.94) (*p* < 0.001) for Cho. The diagnostic accuracy had a sensitivity and specificity of 100% at a cut-off value of 1.02 for Lac+TG, and sensitivity and specificity of 57% and 100%, respectively, at a cut-off value of 0.13 for Cho (Figure 4b).

## 4. Discussion

It is of clinical importance to identify biological markers that can be used for the stratification of patients with liver disease based on disease status as well as prognosis. Our study investigated the potential for using metabolic information obtained from ^1^H-MRS in the cirrhotic liver to evaluate the alterations associated with HCC-related metabolism in conjunction with their association with aging and enzymatic activities. Our data suggest that Lac+TG and Cho levels might be characteristic markers of HCC in vivo. It has been shown that their levels are directly related to cell proliferation, which is linked to carcinogenesis. Our data provides evidence of the diagnostic value of long TE ^1^H-MRS for the evaluation of liver disease and demonstrates that these data can be used for accurate discrimination between LC and HCC, as well as between CLH and HCC. Moreover, LDH and ALP enzyme activities were significantly altered in LC and HCC compared to that in the normal liver; the activities of these enzymes were correlated with the Lac+TG and Cho levels, respectively. Furthermore, these changes in cellular metabolites in patient groups were significantly correlated with age, especially in patients 60–80 years of age. Indeed, our findings revealed that ^1^H-MRS is a reliable, noninvasive tool in patients with LC and HCC, suggesting that long TE ^1^H-MRS may clarify hepatic metabolic changes in HCC that occurs in patients with LC.

In order to assess the reliability of ^1^H-MRS measurements, the present study investigated the possible variability among ^1^H-MRS examination findings by measuring the ICCs. The average ICCs for all the metabolites was > 0.9 (range, 0.980–0.998), showing good reliability for these MRS data. One of the important factors for a reproducible ^1^H-MRS examination is to minimize the respiratory movement, and our study used the single breath-hold technique. Consequently, the ^1^H-MR spectra were successfully obtained with minimal motion artifacts. In addition, the spectral quality of our data was consistent with the reproducible full width at half maximum (FWHM) of water peak in liver patients.

One of the interesting findings in our study was the elevation of the Lac+TG level in HCC compared to that in cirrhotic liver. Meanwhile, high levels of TG were detected in the NC group based on MRS spectral peaks of TG at 0.9 and 2.1 ppm, as well as serum analysis, compared to that in other groups. Although the Lac signal at 1.3 ppm overlapped with large TG resonance in the liver [16] and could not be separated from TG peak in our data, we speculate that Lac contributed to the difference in metabolic patterns between HCC and cirrhotic liver, as supported by a recent in vivo ^1^H-MRS study that showed a disease-specific Lac+TG peak at long TE in patients with non-alcoholic fatty liver disease [9]. Lac accumulates in HCC cells during carcinogenesis and metastasis, making the extracellular pH of the tumor consistently acidic. The increase in anaerobic glycolysis in tumor cells allows metabolism to be directed toward producing more Lac. Consequently, this alteration in metabolism can act as an essential precursor for nucleic acid and phospholipid synthesis, supporting tumor cell proliferation. Moreover, it has been suggested that acidic anaerobic metabolism results in a perturbed environment that may increase the genetic mutation rate leading to tumor initiation [17]. According to several hyperpolarized ^13^C MRS studies [18,19,20,21], [1-^13^C] Lac was significantly elevated in hepatic injuries and HCCs, and significantly higher levels of LDH-A were found in HCC than in the normal liver, suggesting that the elevated levels of Lac in HCC can be attributed to the increased LDH-A enzyme level [18]. In our study, serum LDH activity was also positively correlated with the Lac+TG level in patient groups, suggesting that the elevated LDH level could be associated with abnormal Lac metabolism. From these findings, the Lac+TG level might be a potential quantitative biomarker reflecting disease-specific metabolism in anaerobic conditions during hepatocarcinogenesis in a cirrhotic background.

Another interesting finding in our study was that the Cho level in the HCC group was significantly higher than in the NC and LC groups. Cho is an important constituent in the cell membrane associated with phospholipid metabolism and, therefore, an effective indicator of cell proliferation. Increased choline may encourage rapid proliferation and aggressiveness of HCC cells by activating genes that code for enzymes related to choline metabolism, such as choline kinase-α and phosphatase [17]. Therefore, the high level of Cho in HCC may be considered an indicator of continuous cell proliferation, which is a characteristic of carcinogenesis. An ex vivo study [22] reported an increase in choline-containing compounds relative to lipids in HCC compared to the amount found in background cirrhosis. Additionally, in our study, there was a significant positive correlation between Cho and ALP in patient groups. These findings suggest that Cho may be used as an active biomarker for cellular proliferation associated with HCC, and therefore, quantitative in vivo ^1^H-MRS could provide clinically useful information.

In our results from GEE analysis, potential risk factors for changes in Lac+TG and Cho levels were age, specifically in the 60–80 year range. An important finding was that, as a risk factor for changes in the Lac+TG level, the OR (15.184) of males aged 60–80 years in HCC was two-fold compared to OR (7.465) of males aged 40–50 years. Thus, it is assumed that any patient over 60 years of age, in conjunction with altered Lac+TG and Cho levels, might have a higher risk of developing HCC in cirrhotic liver. According to the ROC analysis for differential diagnosis among LC, CLH, and HCC, we noted a reasonably high diagnostic accuracy of long TE ^1^H-MRS to distinguish HCC from cirrhotic liver based on cut-off values of Lac+TG and Cho. This indicates that ^1^H-MRS may be used as a clinical diagnostic tool.

Our study presents several limitations. First, because we conducted a preliminary study to evaluate that the in vivo metabolite is a promising biomarker to confirm early-stage HCC, follow-up studies will be needed to see if the results equally apply to very early-stage HCC and atypical HCC. Second, as HCC increases in size, it can have a variety of histological components. However, when placing voxels to measure metabolites within HCC, we were careful to minimize the effects of tissue diversity as various histological components, such as well and poorly differentiated tumor cells, may be included inside voxels. Third, when measuring metabolites in the cirrhotic liver parenchyma, it was not possible to determine whether the histological entity of the site contained in the voxel was a regenerative nodule, degenerative nodule, or fibrosis. Therefore, although our results showed that hepatocellular metabolites of Lac+TG and Cho in the cirrhotic liver parenchyma were significantly discriminated from those of early-stage HCC, further studies may be needed to specifically determine whether the in vivo metabolite is a useful biomarker for the differentiation of dysplastic nodule from early or overt HCC. Fourth, we used the residual water peak as an internal reference. The use of water signals is prone to quantification error, caused by different water contents in different tissues. This can potentially be problematic due to different T2 times. In order to prevent the influence of water on different pathologies, an external reference would be necessary for further MRS studies to demonstrate the actual change in metabolites. Fifth, although we used a 3-T MR scanner and longer TE to increase the signal to noise ratio (SNR), spectra containing only noise without any identifiable Cho metabolite peaks were obtained in a few cases. High-field MRI equipment and/or advanced techniques, utilizing Nuclear Overhauser Effect enhancement and proton decoupling, could demonstrate improved SNRs and spectral resolution between MRS peaks [23]. Sixth, metabolite levels in CLH and HCC were obtained from two different MR spectra in successive scans, which was time consuming and led to possible observational management error. Hence, the development of multivoxel two- or three-dimensional chemical shift MRS would be beneficial for future studies. Lastly, the Lac signal at 1.3 ppm could not be separated from the TG peak in our study. Thus, future studies might be needed to separate and quantify spectrally overlapped Lac and TG signals at 1.3 ppm.

In conclusion, we demonstrated that in vivo ^1^H-MRS was useful to quantify hepatic Lac+TG and Cho levels, where higher Lac+TG and Cho values were most likely related to the HCC-related metabolism in the cirrhotic liver. Further, the level of Lac+TG in HCC was highly correlated with older age and LDH levels. Our data suggest that hepatic metabolite quantification obtained from ^1^H-MRS may provide a useful tool for the noninvasive early diagnosis and monitoring of hepatocarcinogenesis in patients with LC.

## Figures and Tables

**Table 1:** Comparison of demographics and serum biochemical analysis among NC, LC without HCC, and LC with HCC groups.

Variables	NC (*n* = 30)	LC without HCC (*n* = 30)	LC with HCC (*n* = 30)	*P*-Value		
				P1	P2	P3
Age (years)	49.0 ± 22.1	54.5 ± 18.9	62.1 ± 23.4	1.000 *	0.064 *	0.064 *
Sex (male/female)	16/14	15/15	15/15	0.957 †		
Serum biochemical analysis						
Aspartate aminotransferase (AST, U/L)	24.0 ± 10.2	42.8 ± 16.9	77.5 ± 39.2	0.119 *	0.002 *	0.671 *
Alanine aminotransferase (ALT, U/L)	33.2 ± 20.0	36.5 ± 14.3	55.1 ± 32.6	0.771 *	0.546 *	0.984 *
Lactate dehydrogenase (LDH, U/L)	385.8 ± 84.7	431.7 ± 110.8	461.8 ± 172.1	0.356 *	0.041 *	0.524 *
Alkaline phosphatase (ALP, U/L)	93.4 ± 43.4	116.8 ± 50.2	147.7 ± 79.6	0.007 *	0.299 *	0.247 *
Glucose (mg/dL)	102.2 ± 20.2	116.9 ± 55.4	121.3 ± 36.1	0.104 *	0.001 *	0.023 *
Triglyceride (TG, mg/dL)	161.6 ± 79.5	127.7 ± 104.3	117.2 ± 41.6	0.586 *	0.141 *	0.867 *
Albumin (g/dL)	4.1 ± 1.8	4.2 ± 1.6	3.9 ± 1.6	0.601 *	0.125 *	0.681 *
Total bilirubin (mg/dL)	0.9 ± 0.4	1.2 ± 0.5	1.5 ± 0.8	0.231 *	0.547 *	0.694 *

NC, normal control; LC, liver cirrhosis; HCC, hepatocellular carcinoma. P1, NCs vs. LC without HCC; P2, NCs vs. LC with HCC; P3, LC without HCC vs. LC with HCC. * One-way analysis of variance (ANOVA) with Tukey’s post-hoc test. † Chi-square test.

**Table 2:** Correlation coefficients between the levels of cellular metabolites and enzymatic activities in a combination of LC, CLH, and HCC groups.

Metabolites	AST (U/L)	ALT (U/L)	LDH (U/L)	ALP (U/L)	Glucose (mg/dL)	TG (mg/dL)	Albumin (g/dL)	Total bilirubin (mg/dL)
Lac+TG (1.3 ppm)	0.030 (*p* = 0.783)	0.074 (*p* = 0.489)	0.299 * (*p* = 0.004)	−0.083 (*p* = 0.439)	0.113 (*p* = 0.289)	−0.143 (*p* = 0.441)	0.046 (*p* = 0.675)	−0.078 (*p* = 0.481)
Cho (3.2 ppm)	0.127 (*p* = 0.237)	0.114 (*p* = 0.179)	0.047 (*p* = 0.657)	0.338 * (*p* = 0.001)	−0.123 (*p* = 0.247)	−0.155 (*p* = 0.404)	0.099 (*p* = 0.362)	−0.153 (*p* = 0.164)
TG (0.9 ppm)	−0.114 (*p* = 0.366)	−0.027 (*p* = 0.830)	0.103 (*p* = 0.410)	0.155 (*p* = 0.214)	0.041 (*p* = 0.743)	−0.051 (*p* = 0.819)	0.114 (*p* = 0.376)	−0.096 (*p* = 0.465)
TG (2.1 ppm)	0.023 (*p* = 0.853)	0.176 (*p* = 0.162)	−0.050 (*p* = 0.688)	−0.083 (*p* = 0.508)	−0005 (*p* = 0.967)	−0.090 (*p* = 0.683)	0.016 (*p* = 0.903)	−0.135 (*p* = 0.302)

* *p* < 0.01. LC, liver cirrhosis without HCC; CLH, cirrhotic liver with HCC; HCC, hepatocellular carcinoma; Lac+TG, lactate+triglyceride; Cho, choline; TG, triglyceride.

**Table 3:** GEE analysis for risk factors associated with metabolic changes of Lac+TG and Cho in LC, CLH, and HCC groups.

Variables	Lac+TG Concentration			Cho Concentration		
	OR	95% CI	*P*-Value	OR	95% CI	*P*-Value
40–50 years old ^a^	1.000	-	-	1.000	-	-
60–80 years old	1.508	1.107–2.053	0.009	1.049	1.006–1.094	0.024
*Interaction effect*						
Female × 40–50 years old × LC group ^b^	1.000	-	-	1.000	-	-
Female × 40–50 years old × CLH group	1.034	0.956–1.119	0.401	1.004	0.971–1.037	0.823
Female × 40–50 years old × HCC group	4.764	3.071–7.389	0.001	1.061	0.994–1.133	0.073
Female × 60–80 years old × LC group	1.115	1.047–1.187	0.001	0.949	0.909–0.990	0.064
Female × 60–80 years old × CLH group	1.133	1.003–1.281	0.045	1.024	0.994–1.055	0.114
Female × 60–80 years old × HCC group	5.265	3.257–8.508	0.001	1.251	1.163–1.346	0.001
Male × 40–50 years old × LC group	1.013	0.935–1.097	0.752	0.99	0.955–1.027	0.606
Male × 40–50 years old × CLH group	1.047	0.956–1.147	0.324	1.035	0.985–1.087	0.172
Male × 40–50 years old × HCC group	7.465	4.481–12.428	0.001	1.183	1.023–1.369	0.054
Male × 60–80 years old × LC group	1.132	1.025–1.250	0.014	1.037	0.978–1.099	0.223
Male × 60–80 years old × CLH group	1.098	1.000–1.206	0.049	1.000	0.970–1.032	0.975
Male × 60–80 years old × HCC group	15.184	7.726–29.839	0.001	1.152	1.060–1.251	0.001

GEE, generalized estimating equations; OR, odds ratio; CI, confidence interval; LC, liver cirrhosis without HCC; CLH, cirrhotic liver with HCC; HCC, hepatocellular carcinoma; Lac+TG, lactate+triglyceride; Cho, choline. ^a^ Reference group for 60–80 years old group. ^b^ Reference group for sex × age × group.

## References

[1] Perman W.H., Balci N.C., Akduman I. (2009). Review of magnetic resonance spectroscopy in the liver and the pancreas. Top. Magn. Reson. Imaging.

[2] Prasad S.R., Wang H., Rosas H., Menias C.O., Narra V.R., Middleton W.D., Heiken J.P. (2005). Fat-containing lesions of the liver: Radiologic-pathologic correlation. Radiographics.

[3] Flemming J.A., Yang J.D., Vittinghoff E., Kim W.R., Terrault N.A. (2014). Risk prediction of hepatocellular carcinoma in patients with cirrhosis: The ADRESS-HCC risk model. Cancer.

[4] Heimbach J.K., Kulik L.M., Finn R.S., Sirlin C.B., Abecassis M.M., Roberts L.R., Zhu A.X., Murad M.H., Marrero J.A. (2018). AASLD guidelines for the treatment of hepatocellular carcinoma. Hepatology.

[5] Bosch F.X., Ribes J., Díaz M., Cléries R. (2004). Primary liver cancer: Worldwide incidence and trends. Gastroenterology.

[6] GBD 2013 Mortality and Causes of Death Collaborators (2015). Global, regional, and national age-sex specific all-cause and cause-specific mortality for 240 causes of death, 1990–2013: A systematic analysis for the Global Burden of Disease Study 2013. Lancet.

[7] Efremidis S.C., Hytiroglou P. (2002). The multistep process of hepatocarcinogenesis in cirrhosis with imaging correlation. Eur. Radiol..

[8] Kim S.Y., An J., Lim Y.S., Han S., Lee J.Y., Byun J.H., Won H.J., Lee S.J., Lee H.C., Lee Y.S. (2017). MRI with liver-specific contrast for surveillance of patients with cirrhosis at high risk of hepatocellular carcinoma. JAMA Oncol..

[9] Kim T.H., Jun H.Y., Kim K.J., Lee Y.H., Lee M.S., Choi K.H., Yun K.J., Jeong Y.Y., Jun C.H., Cho E.Y. (2017). Hepatic alanine differentiates nonalcoholic steatohepatitis from simple steatosis in humans and mice: A proton MR spectroscopy study with long echo time. J. Magn. Reson. Imaging.

[10] Zhang L., Zhao X., Ouyang H., Wang S., Zhou C. (2016). Diagnostic value of 3.0T (1)H MRS with choline-containing compounds ratio (∆CCC) in primary malignant hepatic tumors. Cancer Imaging.

[11] Dagnelie P.C., Leij-Halfwerk S. (2010). Magnetic resonance spectroscopy to study hepatic metabolism in diffuse liver diseases, diabetes and cancer. World J. Gastroenterol..

[12] Xu H., Li X., Yang Z.H., Xie J.X. (2006). In vivo ^1^H MR spectroscopy in the evaluation of the serial development of hepatocarcinogenesis in an experimental rat model. Acad. Radiol..

[13] Erstad D.J., Tanabe K.K. (2017). Hepatocellular carcinoma: Early-stage management challenges. J. Hepatocell. Carcinoma.

[14] Mitchell D.G., Bruix J., Sherman M., Sirlin C.B. (2015). LI-RADS (Liver Imaging Reporting and Data System): Summary, discussion, and consensus of the LI-RADS Management Working Group and future directions. Hepatology.

[15] Hamilton G., Yokoo T., Bydder M., Cruite I., Schroeder M.E., Sirlin C.B., Middleton M.S. (2011). In vivo characterization of the liver fat ^1^H MR spectrum. NMR Biomed..

[16] Ter Voert E.G., Heijmen L., van Laarhoven H.W., Heerschap A. (2011). In vivo magnetic resonance spectroscopy of liver tumors and metastases. World J. Gastroenterol..

[17] Wang J., Zhang S., Li Z., Yang J., Huang C., Liang R., Liu Z., Zhou R. (2011). (1)H-NMR-based metabolomics of tumor tissue for the metabolic characterization of rat hepatocellular carcinoma formation and metastasis. Tumor Biol..

[18] Darpolor M.M., Yen Y.F., Chua M.S., Xing L., Clarke-Katzenberg R.H., Shi W., Mayer D., Josan S., Hurd R.E., Pfefferbaum A. (2011). In vivo MRSI of hyperpolarized [1-(13)C] pyruvate metabolism in rat hepatocellular carcinoma. NMR Biomed..

[19] Moon C.M., Shin S.S., Heo S.H., Lim H.S., Moon M.J., Surendran S.P., Kim G.E., Park I.W., Jeong Y.Y. (2019). Metabolic changes in different stages of liver fibrosis: In vivo hyperpolarized ^13^C MR spectroscopy and metabolic imaging. Mol. Imaging Biol..

[20] Moon C.M., Shin S.S., Lim N.Y., Kim S.K., Kang Y.J., Kim H.O., Lee S.J., Beak B.H., Kim Y.H., Jeong G.W. (2018). Metabolic alterations in a rat model of hepatic ischemia reperfusion injury: In vivo hyperpolarized ^13^C MRS and metabolic imaging. Liver Int..

[21] Moon C.M., Oh C.H., Ahn K.Y., Yang J.S., Kim J.Y., Shin S.S., Lim H.S., Heo S.H., Seon H.J., Kim J.W. (2017). Metabolic biomarkers for non-alcoholic fatty liver disease induced by high-fat diet: In vivo magnetic resonance spectroscopy of hyperpolarized [1-^13^C] pyruvate. Biochem. Biophys. Res. Commun..

[22] Soper R., Himmelreich U., Painter D., Somorjai R.L., Lean C.L., Dolenko B., Mountford C.E., Russell P. (2002). Pathology of hepatocellular carcinoma and its precursors using proton magnetic resonance spectroscopy and a statistical classification strategy. Pathology.

[23] Xu L., Liu B., Huang Y., Liu X., Zhang S.W., Xin X.G., Zheng J.Z. (2013). 3.0 T proton magnetic resonance spectroscopy of the liver: Quantification of choline. World J. Gastroenterol..

